# Sequential Lymphotoxin Beta Receptor and Retinoic Acid Receptor signals regulate cDC2 fates

**DOI:** 10.21203/rs.3.rs-4784425/v2

**Published:** 2025-10-16

**Authors:** Albert A. Nguyen, Logan Fisher, Jennifer S.Y. Ahn, Michelle Zuo, Tyler Park, Anushka Yadav, Miki S. Gams, Kei Haniuda, Dragos C. Dasoveanu, Conglei Li, Lesley A. Ward, Angela A. Wang, Zakieh Tayyebi, Daria Trofimova, Trevor McKee, Cathy L. Mendelsohn, John S. Allingham, Cynthia J. Guidos, Blandine Maître, Jennifer L. Gommerman, Chrysothemis C. Brown

**Affiliations:** 1 Department of Immunology, University of Toronto, Toronto, Ontario, Canada. M5R 3C5; 2 Immunology and Microbial Pathogenesis Program, Weill Cornell Medicine Graduate School of Medical Sciences; New York, NY, USA; 3 Computational and Systems Biology Program, Memorial Sloan Kettering Cancer Center, New York, NY, USA; 4 Immune Oncology Program, Memorial Sloan Kettering Cancer Center; New York, NY, 10065 USA; 5 Program in Cell & Systems Biology, Hospital for Sick Children Research Institute; 6 School of Medicine, The Chinese University of Hong Kong, Shenzhen, China; 7 Computational and Systems Biology Program, Memorial Sloan Kettering Cancer Center, New York, NY, USA; 8 Department of Biomedical and Molecular Sciences, Queen’s University; 9 Department of Laboratory Medicine and Pathobiology, University of Toronto, Toronto, Ontario, Canada. M5R 3C5; 10 Department of Pathology and Cell Biology, Columbia University; 11 Université de Strasbourg, Etablissement Français du Sang (EFS) Grand Est, BPPS UMR_S 1255, Strasbourg, France; 12 Department of Pediatrics, Memorial Sloan Kettering Cancer Center; New York, NY, 10065, USA; 13 Howard Hughes Medical Institute, Memorial Sloan Kettering Cancer Center; New York, NY, 10065, USA

## Abstract

Type II conventional dendritic cells (cDC2) are functionally and phenotypically heterogenous. Previous work in mice and humans identified two cDC2 subsets (cDC2A and cDC2B) and a monocytic DC3 subset. However, the microenvironmental cues governing their distinct differentiation pathways remain unclear. Here we delineate murine cDC2 lineage relationships and the sequential signals required for cDC2A maintenance. We show that cDC2s, arising from the CLEC9A^+^ cDC progenitor, encompass T-bet-expressing cDC2A and two cDC2B subsets distinguished by MGL2 expression, with monocytic DC3 exhibiting transcriptional overlap with *Mgl2*^−^ cDC2B. Among these subsets, T-bet^+^ cDC2A dominate the spleen where they require cell-intrinsic retinoic acid (RA) signaling to sustain their differentiation via Notch signals. Lymphotoxin beta receptor signaling on splenic cDC2s limits F-actin content retaining cDC2 at sites of retinol delivery. In summary, these data establish the developmental and transcriptional relationships between diverse cDC2 subsets and identify signals that regulate their prevalence in specific lymphoid tissues.

Conventional dendritic cells (cDCs) encompass two developmentally and functionally distinct subsets, XCR1^+^ cDC1 and cDC2, the latter distinguished by expression of either SIRPα or CD11b. While cDC1s are required for cytotoxic CD8^+^ T cell priming, cDC2s have been implicated in CD4^+^ T cell mediated immunity, most notably Th2 ^1, 2^ and Th17 ^3, 4, 5^ differentiation. In contrast to the cDC1 lineage, cDC2s are transcriptionally and functionally heterogenous. Previous studies identified two major cDC2 subsets, variably termed cDC2A and cDC2B ^6^, or DC2 and DC3 ^7^. A comparative analysis of cDC2A/B and DC2/3 transcriptomes demonstrated that cDC2A are analogous to DC2 whereas cDC2B correspond to DC3, with DC3 arising from a Ly6C^+^ monocyte-DC progenitors (MDP) ^7^. This is at odds with the finding that cDC2A and cDC2B arise from Clec9a^+^ classical DC progenitors, with fate specification occurring in the bone marrow (BM) ^8, 9^. One possibility, suggested by a single cell RNA sequencing (scRNA-seq) analysis of DCs, is that cDC2B and DC3 co-exist as two transcriptionally and ontogenically distinct DC subsets ^9^. Adding to the complexity, a recent study suggested that ESAM^+^ cDC2s, previously thought to reflect cDC2A, encompassed two phenotypically and ontogenically distinct subsets of DC2s – CD11b^lo^ESAM^+^ cDC2A and CD11b^hi^ ESAM^+^ DC2 – with the latter representing the dominant DC2 subset within the spleen ^10^. A better understanding of the distinguishing transcriptional programs and features of cDC2A, cDC2B and DC3 is key to unravelling their unique functions.

The differential abundance of cDC2A vs cDC2B across tissues and lymph nodes suggests that, following their specification in the BM, additional peripheral cues shape the identity of these subsets and/or the recruitment of pre-specified cDC2 progenitor cells ^8^. Such cues are not well described yet are critical to our understanding of how secondary lymphoid tissues can support distinct immunological outcomes. For example, cDC2A are dependent on Notch signaling, and expression of Notch2 in CD11c^+^ cells is required for splenic Tfh and intestinal Th17 responses ^3, 4, 11^. On the other hand, cDC2B are dependent on *Klf4* ^2^, and conditional deletion of *Klf4* in CD11c^+^ cells impairs Th2 responses, resulting in poor immunity against *S. mansoni* challenge in the skin, and house dust mite challenge in the lung ^2^. Understanding tissue-specific cues that regulate cDC2 cell fates is thus key to modulating CD4^+^ T cell immune responses.

In this study, we investigated the full spectrum of cDC2 heterogeneity and the environmental cues that shape cDC2 fates in lymphoid tissues. We show that cDC2s encompass cDC2A and two subsets of cDC2B distinguished by expression of MGL2, with *Mgl2*^−^ cDC2B displaying transcriptional overlap with previously described monocytic DC3. While cDC2A and cDC2B subsets were derived from CLEC9A^+^ common DC progenitors (CDP), their relative proportions varied across secondary lymphoid tissues, indicating a role for environmental cues in regulating the balance between these subsets. Using expression of T-bet by cDC2A to selectively disrupt candidate tissue-specific signals, we identified sequential roles for the TNF superfamily member receptor lymphotoxin beta receptor (LTβR) and retinoic acid (RA), a metabolic derivative of dietary Vitamin A, in promoting and maintaining cDC2A differentiation, respectively.

## Lineage tracing and scRNA-seq define cDC2A, cDC2B and DC3 subsets in lymphoid tissues

**Fig. 1:**
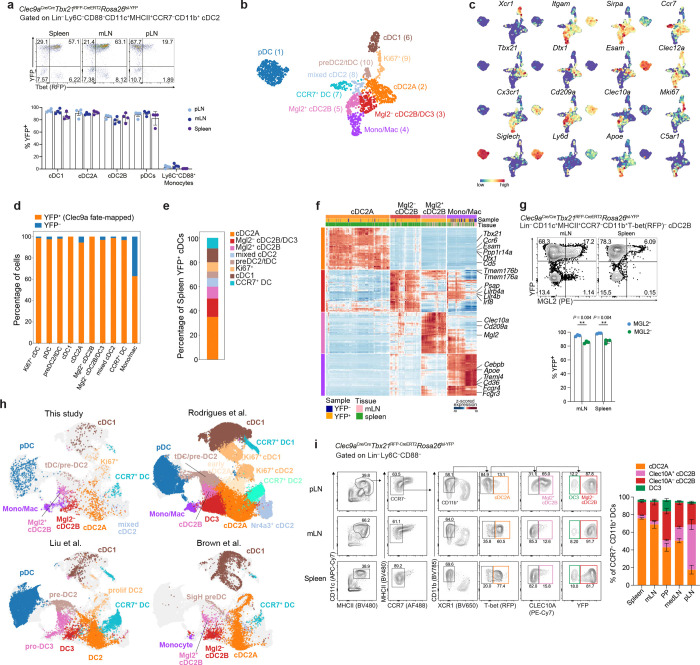
Tissue environmental cues determine the balance of cDC2A and cDC2B subsets. **a**, Representative flow cytometry analysis of cDC2 from *Clec9a*^Cre/Cre^*Tbx21*^RFP-CreERT2^*R26*^lsl-YFP^ mice (top), and summary bar graphs (bottom) demonstrating frequency of YFP labeled cells for each indicated cell subset and lymphoid tissue (*n* = 4 mice per group). **b**, Uniform manifold approximation and projection (UMAP) visualization of YFP^+^ and YFP^−^ Lin (TCRβ, CD19, NK1.1)^−^ MHCII^+^ cells from mLN and spleen of *Clec9a*^Cre/Cre^*Tbx21*^RFP-CreERT2^*R26*^lsl-YFP^ mice profiled by scRNA-seq, colored by cluster. **c**, UMAP overlaid by expression of indicated genes. **d**, Bar graph showing proportion of YFP^+^ (*Clec9a*-fate mapped) and YFP^−^ cells within each cluster. **e,** Bar graph showing proportion of cDC clusters identified in (b) among splenic YFP^+^ cDCs. **f**, Heat map showing scaled, imputed expression of top 70 DEGs (one versus the rest, fold change (FC) > 1.5, adjusted P < 0.01) for indicated CCR7^−^ cDC2 clusters. **g**, Representative flow cytometry analysis of splenic and mLN T-bet(RFP)^−^ cDC2s from *Clec9a*^Cre/Cre^Tbx21^RFP-CreERT2^*R26*^lsl-YFP^ mice (left), and summary bar graphs (right) demonstrating frequency of YFP labeled cells for MGL2^+^ vs MGL2^−^ cDC2B (*n* = 3 mice per group). **h**, UMAP visualization of dendritic cells from 1c, Rodrigues et al., Liu et al., and Brown et al. colored by original cell type annotation or cell type annotation from Extended Data Fig. 1f (Rodrigues et al.). **i**, Representative flow cytometry demonstrating gating strategy for identification of cDC2A, cDC2B and DC3 subsets in lymph nodes and spleen of *Clec9a*^Cre/Cre^*Tbx21*^RFP-CreERT2^*R26*^lsl-YFP^ mice (left), and summary graph showing cDC2 subset proportions in indicated lymphoid tissues (right) (*n* = 3 mice). Data in **a**, **g** and **i** are representative of 2 independent experiments. Data are mean ± s.e.m., each symbol represents an individual mouse. One-way ANOVA (a) or unpaired two-tailed t test (g). **P < 0.01.

The aim of our study was to identify the signals that regulate cDC2 heterogeneity within secondary lymphoid tissues. Since differences in cDC2 composition could reflect homing of distinct progenitors to these sites, or distinct tissue cues driving differentiation of a common progenitor into separate cDC2 lineages, we first rigorously validated previously proposed cDC2 subsets and their ontogeny using lineage-tracing and gene expression. To distinguish cDC2A, cDC2B and DC3 subsets, we generated *Clec9a*^Cre/Cre^ *Tbx21*^RFP-CreERT2^*R26*^lsl-YFP^ mice in which descendants of CLEC9A^+^ CDP are labeled with YFP, and cDC2A can be distinguished from cDC2B/DC3 by expression of RFP ^11^. Analysis of splenic cDC2 subsets revealed that ~95% of T-bet^+^ cDC2A and ~ 85% of T-bet^−^ cDC2 were YFP^+^ with similar proportions of fate-mapped cells observed across different lymphoid tissues (Fig. 1a and **Extended Data Fig. 1a**). By contrast, CD88^+^Ly6C^+^ monocytes exhibited negligible YFP labeling. To determine if the unlabeled T-bet^−^ cDC2s represented monocytic DC3, we examined expression of CD16/CD32, a proposed marker for DC3 ^21^. Surprisingly, we observed increased proportions of CD16/32^+^ cells among YFP^*+*^ T-bet^−^ cDC2Bs relative to their YFP^−^ counterparts (**Extended Data Fig. 1b**). To better identify markers that would distinguish ontogenically distinct cDC2B and DC3 subsets, we performed scRNA-seq analysis of YFP^+^ and YFP^−^ Lin^−^ (TCRβ^−^CD19^−^SiglecF^−^NK1.1^−^) MHCII^+^ cells isolated from spleen and gut-draining mesenteric lymph nodes (mLN) of *Clec9a*^Cre/Cre^ *Tbx21*^RFP-CreERT2^*R26*^lsl-YFP^ mice (**Extended Data Fig. 1c**). Cells were labelled with oligonucleotide-tagged antibodies to allow retrospective identification according to their tissue of origin and ontogeny. Unsupervised clustering alongside analysis of canonical DC marker genes identified a cluster of *C5ar1* and *Apoe*-expressing monocyte/macrophages (cluster 4), plasmacytoid DCs (pDCs; cluster 1), CCR7^−^ cDC1s (cluster 6), CCR7^+^ ‘migratory’ DCs (cluster 7) and pre-DC2/transitional DCs (cluster 10). CCR7^−^CD11b^+^ DC2s, the focus of this study, spanned one cluster of *Tbx21*^+^*Esam*^+^ cDC2A (cluster 2) and two clusters expressing *Cd209a*, *Cx3cr1* and *Clec12a*, signature markers for both cDC2B and DC3 ^6, 7, 9^ (Fig. 1b, c). *Esam*^+^ cDC2s (cluster 2) exhibited uniformly high levels of *Itgam* (Fig. 1c), encoding CD11b, and ~95% of these cells were YFP^+^ (Fig. 1d), confirming their cDC2A identity. This cDC2A cluster represented the dominant cDC2 subset among YFP-labeled splenic cDCs (Fig. 1e), consistent with our earlier flow cytometry analysis.

Analysis of differential gene expression alongside signature cDC2 genes demonstrated that cluster 5 represented *Mgl2*^*+*^*Clec10a*^*+*^ cDC2Bs that were universally labeled by YFP (Fig. 1d, f). Cells in cluster 3 expressed higher levels of genes previously suggested to distinguish DC3 from cDC2B, including *Lilr4b* and *Lilrb4a* (Fig. 1f) ^9^. However, rather than clusters 3 and 5 representing ontogenically distinct DC3 and cDC2B subsets, analysis of *Clec9a*-fate mapping revealed that >95% of cluster 3 cells were also YFP^+^ (Fig. 1d). These findings suggested that cDC2B cells comprise two transcriptionally distinct subsets or cell states, distinguished by *Mgl2* or *Clec10a* expression, consistent with the original description of splenic cDC2B cells ^6^. Cell surface protein analysis confirmed co-expression of MGL2 and CLEC10A by a subset of cDC2B (**Extended Data Fig. 1d**). In line with our scRNA-seq analysis, close to 100% of MGL2^+^ and ~85% MGL2^−^ cDC2B were descended from *Clec9a*^+^ DC progenitors (Fig. 1g).

We wondered whether the YFP^−^T-bet^−^MGL2^−^ cDC2s represented monocytic DC3. In support of this, expression of *Zbtb46*, a transcription factor that distinguishes cDCs from monocytes, was lower in YFP^−^ cells compared to their YFP^+^ counterparts (**Extended Data Fig. 1d**, **e**). To definitively establish the overlapping and distinct transcriptional phenotypes between cDC2B and DC3, we integrated our scRNA-seq dataset with publicly available data from Brown et al., Liu et al., and Rodrigues et al ^6, 7, 9^. Reanalysis of the scRNA-dataset from Rodrigues et al. confirmed the presence of two *Cx3cr1*^*+*^ cDC2 clusters (**Extended Data Fig. 1f**, **g** and **Supplementary Table 1**) with differential expression of DC3 genes (**Extended Data Fig. 1h**) and differential *Cd300c* fate-mapping (**Extended Data Fig. 1i**). We thus annotated these cells as DC3 or cDC2B respectively, in line with their annotation in the original study ^9^. Visualization of the integrated data (Fig. 1h) demonstrated that cells identified as cDC1, cDC2A, CCR7^+^ DCs and pDCs in all 4 studies localized together in their respective regions of the UMAP, confirming that the integration method accurately maps the same type of cells with similar transcriptional programs. Consistent with previous analyses ^7^, DC2 cells mapped to cDC2A. Intriguingly, cells annotated as DC3 by Rodrigues et al. or Liu et al. mapped to the cluster of *Mgl2*^−^ cDC2B defined by Brown et al. and further validated in this study, whereas cells annotated as cDC2B (Rodrigues et al.) or pro-DC3 (Liu et al.) aligned with *Mgl2*^+^ cDC2B. Collectively these findings demonstrate that cDC2B encompass two transcriptionally distinct cell states or subsets that can be distinguished by expression of either MGL2 or CLEC10A, and that previously defined monocytic DC3 are transcriptionally overlapping with *Mgl2*^−^ cDC2B cells.

Together, these data establish the landscape of cDC2s, identifying one population of T-bet^+^ cDC2A and two subpopulations of cDC2B (**Extended Data Fig. 1j**). This provides a framework for interrogating the tissue-specific cues that shape cDC2 identity using robust markers to distinguish cDC2A and cDC2B subsets.

## Environmental cues determine the balance of cDC2 subsets within lymphoid tissue

Having established the utility of *Clec9a* lineage tracing alongside T-bet and either MGL2 or CLEC10A expression to delineate the full spectrum of cDC2A, cDC2B and DC3 subsets, we next determined how cDC2 composition varies across different lymphoid tissues. To this end, we performed flow cytometry analysis of DC subsets across spleen, skin-draining peripheral lymph nodes (pLN), lung-draining mediastinal lymph nodes (medLN), mLN and Peyer’s patches (PP) of *Clec9a*^Cre/Cre^ *Tbx21*^RFP-CreERT2^*R26*^lsl-YFP^ mice. Within the spleen and mLN, cDC2As represented the majority (~65–80%) of CCR7^−^CD11b^+^ DCs (Fig. 1i). By contrast within the pLN, cDC2A comprised a minor fraction (< 20%) of CCR7^−^CD11b^+^ DCs with increased proportions of CLEC10A^+^ cDC2B. The frequency of CLEC10A^−^ cDC2B and DC3 subsets were similar across different lymphoid tissues.

Overall, these data suggest that environmental cues within the lymphoid tissue determine the relative abundance of cDC2s subsets with enrichment of cDC2A in spleen and gut lymph nodes relative to skin-draining lymph nodes.

## RAR signals are required for the maintenance of cDC2A in vivo.

We next investigated the environmental signals that determine relative abundance of cDC2A vs cDC2B. Given the dominance of cDC2A in the spleen, we focused on this organ. Within the spleen, cDC2 are localized to the marginal zone bridging channels (MZBC), a prime location for the accumulation of blood-borne antigens and macromolecules ^12^. A previous study demonstrated a role for Vitamin A in maintaining Notch2-dependent ESAM^+^ splenic cDC2 ^13^. To systemically impair RAR signaling in C57/Bl6 mice *in vivo*, we administered a Vitamin A Deficient (VAD) diet to pregnant dams from E14, and weaned mice onto a VAD diet. This regime ensures long-term liver stores of maternally-derived retinol are gradually depleted in the pups as they matured ^14^. As previously reported ^13^, flow cytometry analysis revealed that 8–10-week-old VAD mice had reduced splenic cDC2/cDC1 ratios compared with control diet-fed mice (**Extended Data Fig. 2a**).

Among cDC2s, the frequency of ESAM^+^ cells and their level of ESAM expression were reduced in VAD mice compared to controls (**Extended Data Fig. 2b**, **c**). Imaging Mass Cytometry (IMC) analysis of spleen sections revealed a loss of DCIR2^+^ cDC2^15^ in the MZBC of VAD mice compared to controls despite similar follicle size (**Extended Data Fig. 2d-e**). In addition, supplementation of VAD diet with RA over a 10-day period resulted in elevated cDC2 frequency and numbers, and increased ESAM expression on cDC2 compared to vehicle control treated mice (**Extended Data Fig. 2f-h**). Consistent with previous reports ^13, 16, 17^, manipulation of RA levels did not alter cDC1 numbers in either the spleen or PPs (**Extended Data Fig. 2i-k**), despite a reduction in cDC2 in VAD animals. Collectively these data demonstrate that RAR signaling is required to maintain ESAM-expressing MZBC-resident cDC2 in the spleen and CD11b^+^ cDC2 in the PP.

**Fig. 2:**
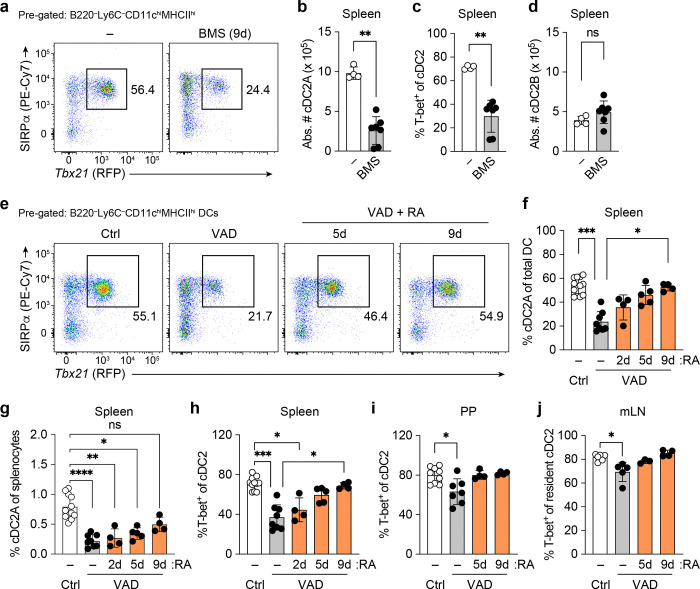
RAR signals in vivo are required for the maintenance of the cDC2A subset. **a**, Representative flow cytometry plots of spleen cDC2A (Sirpa^+^T-bet^+^) frequencies in *Tbx21*^RFP-Cre^ mice treated *i.p.* with BMS493 or a DMSO vehicle control over a 9-day regimen. DC were pre-gated for Live B220^−^Ly6C^−^CD11c^hi^MHCII^hi^ cells. **b-d,** Compiled data of **a,** showing numbers (**b**) and frequencies (**c**) of cDC2A and numbers of cDC2B (**d**), DMSO (*n* = 4) and BMS (*n* = 7). **e,** Representative flow cytometry plots of spleen cDC2A frequencies of *Tbx21*^RFP-Cre^ animals treated with a Vitamin A control or VAD diet and VAD diet animals treated with RA *i.p.* for 5 days and 9 days. **f, h,** Compiled data of **e,** showing cDC2A frequencies of total DC (**f**), cDC2A frequencies of total splenocytes (**g**), and T-bet expression of Sirpa^+^ cDC2 (**h**), Ctrl + DMSO (*n* = 11), VAD + DMSO (*n* = 8), VAD + RA (2d) (*n* = 4), VAD + RA (5d) (*n* = 5), VAD + RA (9d) (*n* = 4)). **i,** Compiled data showing T-bet expression in PP cDC2, Ctrl + DMSO (*n* = 9), VAD + DMSO (*n* = 7), VAD + RA (5d) (*n* = 4), VAD + RA (9d) (*n* = 4)). **j,** Compiled data showing T-bet expression in mLN resident cDC2, Ctrl + DMSO (*n* = 6), VAD + DMSO (*n* = 5), VAD + RA (5d) (*n* = 3), VAD + RA (9d) (*n* = 4)). Data in **b-d**, **i**, and **j** are representative of two independent experiments and **f-h** are representative of four independent experiments. Statistics were measured by two-sided Mann-Whitney U test in **b**, **c**, and **d** (^**^P<0.01) and Kruskal-Wallis test with Dunn’s multiple-comparisons test (*P<0.05, *P<0.01, ***P<0.001, and ****P<0.0001) in **f**, **g**, **h**, **i** and **j**. Error bars represent the mean ± s.d, each symbol represents an individual mouse.

The vast majority of gut-associated cDC2, including those within the PPs, do not express notable levels of ESAM despite the overall abundance of T-bet^+^ cDC2A (**Extended Data Fig. 3a, b**). We therefore turned to *Tbx21*^RFP-Cre^ mice to specifically query the role of RA in the maintenance of cDC2A in the spleen, PP and mLN. We first assessed cDC subsets in adult *Tbx21*^RFP-Cr*e*^ mice whose RAR signaling was acutely inhibited with a pan-RAR small molecule antagonist BMS-493 (gating strategy depicted in **Extended Data Fig. 3c)**. BMS-treated animals exhibited significantly reduced splenic cDC2A frequency and numbers compared to DMSO-treated controls, while cDC2B numbers were unchanged across treatment groups (Fig. 2a-d). Similarly, in VAD *Tbx21*^RFP-Cre^ mice, 9 days of RA supplementation in adulthood resulted in a significant elevation in the frequency of cDC2A compared with vehicle control supplementation (Fig. 2e-j). Taken together, these findings show that RA signaling regulates the accumulation of T-bet^+^ cDC2A in the spleen, PP and mLN.

## Maintenance of T-bet^+^ cDC2A requires cell-intrinsic RAR signaling.

RA impacts multiple cell types and could thus promote T-bet expression in cDC2 in a direct or indirect manner ^18^. Moreover, a VAD diet impacts splenic cellular composition ^19^. Indeed, analysis of splenic immune cells in VAD vs control mice revealed a reduction in CD4^+^ and CD8^+^ T cells and an increase in neutrophils (**Extended Data Fig. 4a-g**). To assess a cDC2A-intrinsic role for RAR signaling, we utilized dn*Rara*^lsl/lsl^ mice which express a dominant-negative form of the retinoic acid receptor RARα (dnRAR) downstream of a *lox*P-flanked STOP cassette^20^, thereby disrupting ligand dependent RAR signaling ^21^.

**Fig. 3:**
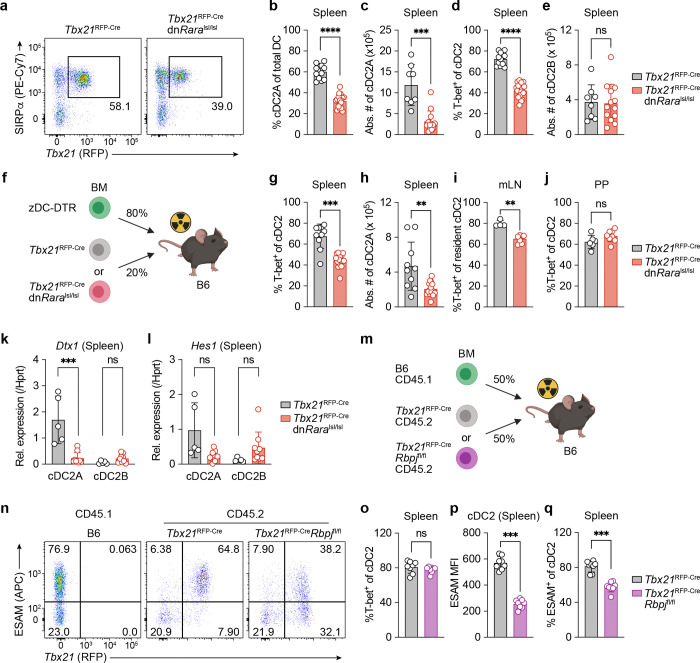
cDC-intrinsic RAR signaling is required for the maintenance of cDC2A. **a**, Representative flow cytometry plots of spleen cDC2A frequencies in animals (right) vs. littermate controls (left). **b-e,** Compiled data of **a,** showing frequencies (**b**) and numbers (**c**) spleen cDC2A, frequency of T-bet^+^ cells among cDC2 (**d**), and numbers of cDC2B (**e**). *Tbx21*^RFP-Cre^ (*n* = 12) and *Tbx21*^RFP-Cre^dn*Rara*^lsl/lsl^ (*n* = 18) for b and d. *Tbx21*^RFP-Cre^ (*n* = 8) and *Tbx21*^RFP-Cre^dn*Rara*^lsl/lsl^ (*n* = 13) for c and e. **f,** Schematic of zDC-DTR mixed BM chimera approach to study cDC2A-intrinsic loss of RAR signaling *in vivo*. **g, h,** Compiled data showing frequency showing T-bet^+^ cells among cDC2 (**g**) and numbers of cDC2A (**h**) in zDC-DTR chimeras containing *Tbx21*^RFP-Cre^ (*n* = 10) or *Tbx21*^RFP-Cre^dn*Rara*^lsl/lsl^ donor BM (*n* = 15). **i, j,** Compiled data showing T-bet expression in (**i**) mLN-resident CD11c^hi^MHCII^int^ cDC2 and (**j**) PP-resident cDC2 in zDC-DTR chimeras containing *Tbx21*^RFP-Cre^ or *Tbx21*^RFP-Cre^dn*Rara*^lsl/lsl^ donor BM. *Tbx21*^RFP-Cre^ (*n* = 4) and *Tbx21*^RFP-Cre^dn*Rara*^lsl/lsl^ (*n* = 7) for i and *Tbx21*^RFP-Cre^ (*n* = 6) and *Tbx21*^RFP-Cre^dn*Rara*^lsl/lsl^ (*n* = 8) for j. **k, l**, Relative expression of (**k**) *Dtx1* and (**l**) *Hes1* measured by qPCR in spleen cDC2A vs. cDC2B from zDC-DTR chimeras containing *Tbx21*^RFP-Cre^ (*n* = 5) or *Tbx21*^RFP-Cre^dn*Rara*^lsl/lsl^ (*n* = 9) donor BM. **m,** Schematic of mixed competitive BM chimera approach to study cDC2A-intrinsic loss of Notch signaling in vivo. **n,** Representative flow cytometry plots showing ESAM and T-bet RFP expression from CD45.1^+^ B6 and CD45.2^+^ *Tbx21*^RFP-Cre^ or *Tbx21*^RFP-Cre^*Rbpj*^fl/f1^ donors in chimeras. **o-q**, Compiled data of **n**, showing T-bet expression (**o**), ESAM MFI (**p**), and ESAM expression (**q**) in CD45.2^+^ cDC2 containing *Tbx21*^RFP-Cre^ or *Tbx21*^RFP-Cre^*Rbpj*^fl/f1^ donor-derived cells (*n* = 8 per group). Data in **b** and **d** are representative of six independent experiments; **c** and **e** are representative of four independent experiments; **i**, **j**, **k**, **l**, and **o**-**q** are representative of two independent experiments. Statistics were measured by two-sided Mann-Whitney U test in **b**, **c**, **d**, **e**, **g**, **h**, **i**, **j**, **k**, **l**, **o**, **p**, **q** (ns, *P*>0.05, ^**^P<0.01, ^***^P<0.001, and ^****^P<0.0001). Error bars represent the mean ± s.d, each symbol represents an individual mouse.

Given the recent identification of a pre-specified T-bet^+^ cDC2A (‘pre-DC2A’) in the BM ^8^, we first investigated whether inhibition of RA signaling would impact cDC2A development at this early stage. To test this, we generated *Zbtb46*^Cre^dn*Rara*^lsl/lsl^ mice, thereby blocking RAR signaling within *Zbtb46*-expressing pre-cDCs. We observed comparable frequencies of SiglecH^+^ pre-cDC2A and SiglecH^−^ pre-cDC2B subsets in *Zbtb46*^Cre^dn*Rara*^lsl/lsl^ mice relative to littermate controls (**Extended Data Fig. 5a**, **b**), suggesting that RAR signaling is not essential for early specification decisions for the pre-cDC2A lineage in the BM. By contrast, *Tbx21*^RFP-Cre^dn*Rara*^lsl/lsl^ mice exhibited reduced frequencies and numbers of cDC2A in the spleen with no observable changes in cDC2B (Fig. 3a-e), consistent with our earlier findings in VAD mice. Interestingly, although T-bet^+^ cDC2A are relatively infrequent in the pLN, there was no further reduction in pLN cDC2A frequencies driven by expression of the dnRAR construct (**Extended Data Fig. 5c**, **d**), suggesting that the requirements for RAR signaling for maintaining cDC2A vary across secondary lymphoid tissues.

Since T-bet s expressed in many cell types, we next determined the cell intrinsic role of RAR signaling in cDC2As. To do this, we generated BM chimera mice with a mixture of *Zbtb46*^iDTR^ (80%) BM + either 20% *Tbx21*^RFP-Cre^dn*Rara*^lsl/lsl^ or *Tbx21*^RFP-Cre^ littermate control donor BM (so-called “zDC” chimeras). Post BM-engraftment, we treated chimeras with diphtheria toxin (DT) for 10 days to ablate *Zbtb46*-expressing cDCs and allow for re-seeding of the cDC compartment by dnRAR-expressing vs WT precursor cells (Fig. 3f). Expression of dnRAR specifically in T-bet^+^ cDC2A resulted in a reduction in cDC2A frequencies and numbers within the spleen and mLN (Fig. 3g-i). However, we did not observe any differences in PP T-bet^+^ cDC2A frequencies (Fig. 3j). These findings were further supported by results from 50:50 competitive BM chimeras whereby splenic dnRAR-expressing cDC2 displayed reduced ESAM expression and impaired fitness compared to control cDC2 (**Extended Data Fig. 5e-h**). In contrast, cDC2B were unaffected by the expression of the dnRAR construct (**Extended Data Fig. 5i**).

These data demonstrate a cell-intrinsic role for RAR in the regulation of splenic and mLN, but not PP cDC2A, revealing tissue-specific differences in cDC2A differentiation.

## An RAR-Notch signaling circuit maintains cDC2A identity in the spleen

Previous literature has shown that Notch signaling is a key regulator of cDC2A development ^3, 4, 6^. Analysis of the canonical Notch-dependent target genes *Dtx1* and *Hes1* revealed reduced expression in cDC2A expressing the dnRAR construct compared to controls, suggesting that RAR signaling contributes to the regulation of Notch signaling in cDC2A. By contrast, cDC2B exhibited a trend towards an increase in *Hes1* expression (Fig. 3k, l), suggesting that under conditions of cDC2A reduction, cDC2B may abnormally access Notch ligands including DLL1.

Although disrupted Notch2 signaling impairs cDC2A numbers ^3, 4^, whether these signals are required for cDC2A lineage specification and/or maintenance of cDC2A identity is unclear. To address this question, we generated competitive chimeras with a 50:50 mix of *Tbx21*^RFP-Cre^*Rbpj*^fl/fl^ and CD45.1 donor BM (Fig. 3m). While we did not observe differences in the proportion of T-bet^+^ cDC2A in this setting (Fig. 3n, o), we noted a significant reduction in ESAM expression among *Rbpj*-deficient cDC2A (Fig. 3p, q). Together, these results demonstrate a role for Notch signaling in T-bet^+^ cDC2A for maintaining ESAM expression in splenic cDC2A.

## LTβR signaling precedes RAR signaling to promote splenic cDC2A development

**Fig. 4:**
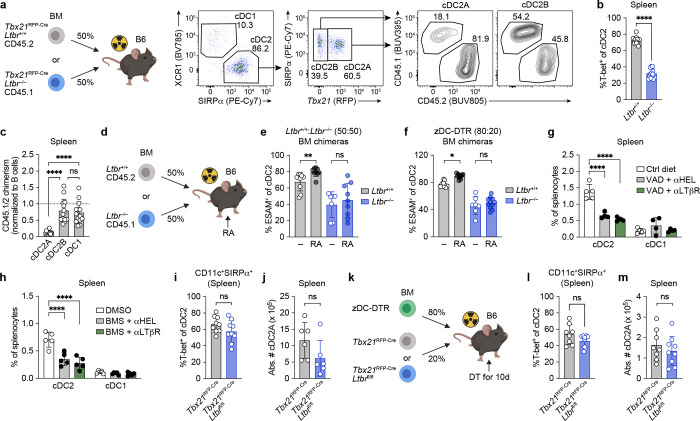
LTβR signaling precedes RAR signaling to promote splenic cDC2A development. **a**, Schematic of competitive (50:50) mixed BM chimeras employing CD45.1 *Tbx21*^RFP-Cre^*Ltbr*^−/−^ and *Tbx21*^RFP-Cre^*Ltbr*^+/+^ CD45.2 cells and representative flow cytometry plots that compare chimerism in the cDC2A and cDC2B compartments. **b, c** Compiled data of **a**, showing T-bet expression (b) and CD45.1(KO)/CD45.2(WT) chimerism (c) of splenic cDC subsets (adjusted according to B cell chimerism) (*n* = 14 per group). **d,** Schematic of competitive (50:50) mixed BM chimeras employing CD45.1 *Ltbr*^−/−^ and CD45.2 *Ltbr*^+/+^ cells. **e,** % ESAM expression on WT and *Ltbr*^−/−^ cDC2 from competitive (50:50) mixed BM chimeras. *Ltbr*^**+/+**^ **+** DMSO (*n* = 9), *Ltbr*^**+/+**^ **+** RA (*n* = 9), *Ltbr*^**−/−**^ **+** DMSO (*n* = 7), *Ltbr*^**−/−**^ **+** RA (*n* = 9). **f,** Frequency of ESAM^+^ cDC2A among cDC2 derived from *Ltbr*^+/+^ (left) or *Ltbr*^−/−^ (right) donor BM after 10 days of DT-ablation in mixed *Zbtb46*^DTR^ 80:20 (*Zbtb46*^DTR^:CD45.1^+^) or (*Zbtb46*^DTR^:CD45.1^+^*Ltbr*^−/−^) BM chimeras that were either treated with RA or DMSO vehicle control. *Ltbr*^**+/+**^ **+** DMSO (*n* = 9), *Ltbr*^**+/+**^ **+** RA (*n* = 11), *Ltbr*^**−/−**^ **+** DMSO (*n* = 6), *Ltbr*^**−/−**^ **+** RA (*n* = 11). **g, h,** Compiled frequencies of cDC2 in VAD animals (g) or BMS493-treated animals (h) that were treated with anti-LTβR agonist antibodies (4H8) or isotype control. Ctrl (*n* = 4), VAD + αLTβR (*n* = 5), VAD + αHEL (*n* = 5) for g; *n* = 5 per group for h. **i, j,** Compiled frequencies (**i**) and numbers (**j**) of splenic T-bet^+^ cDC2A in BM chimeras reconstituted with either *Tbx21*^RFP-Cre^ littermate control or *Tbx21*^RFP-Cre^*Ltbr*^fl/fl^ donor cells (*n* = 9 per group for i, *n* = 7 mice per group for j). **k,** Schematic of zDC-DTR mixed BM chimera approach to study cDC2A-intrinsic loss of LTβR signaling *in vivo*. **l, m,** Compiled frequencies (**l**) and numbers (**m**) of splenic T-bet^+^ cDC2A after 10 days of DT-ablation in mixed 80:20 BM chimeric mice whereby 80% of the BM was derived from *Zbtb46*^iDTR^ mice and 20% was derived from either *Tbx21*^RFP-Cre^ littermate controls (*n* = 8) or *Tbx21*^RFP-Cre^*Ltbr*^fl/fl^ mice (*n* = 9). Data in **b**, **c**, **e**, **f**, **j**, **l** and **m** are representative of two independent experiments; **g** and **h** are representative of one experiment each; **i** is representative of three independent experiments; and **j** is representative of two independent experiments. Statistics were measured by two-sided Mann-Whitney U test (ns, *P*> 0.05, *P<0.05, ^**^P<0.01, and ^****^P<0.0001) in **b**, **e**, **f**, **i**, **j**, **l**, and **m**, Kruskal-Wallis Test with Dunn’s multiple-comparisons test (ns, *P*>0.05 and ^****^P<0.0001) in **c**, and two-way ANOVA with Sidak’s multiple-comparisons test (ns, *P*>0.05 and ^****^P<0.0001) in **g** and **h**. Error bars represent the mean ± s.d, each symbol represents an individual mouse.

cDC-intrinsic expression of the TNF superfamily receptor member LTβR is required for the maintenance of splenic Notch2-dependent ESAM^+^ cDC2 numbers via provision of LTαβ ligand by B cells and ILC3 ^22, 23^. However, whether LTβR is required for cDCA vs cDC2B maintenance is not known. To answer this, we generated CD45.1 *Tbx21*^RFP-Cre^*Ltbr*^*−/−*^ mice and generated 50:50 mixed bone marrow chimeras with CD45.1 *Tbx21*^RFP-Cre^*Ltbr*^*−/−*^ and CD45. 2 *Tbx21*^RFP-Cre^*Ltbr*^*+/+*^ control mice (Fig. 4a). While cDC1 and cDC2B were derived equally from both *Ltbr*^+/+^ and *Ltbr*^−/−^ precursor cells (Fig. 4a), *Ltbr*^+/+^ splenic cDC2A had a strong competitive advantage over *Ltbr*^−/−^ cDC2A (Fig. 4b, c), confirming a critical role for LTβR signaling in regulating cDC2A abundance.

Next, to determine if LTβR signaling occurs upstream or downstream of RAR signaling, we subjected *Ltbr*^+/+^:*Ltbr*^−/−^ chimeras to daily RA supplementation (Fig. 4d). We observed an increase in the frequency of WT but not *Ltbr*^−/−^ ESAM^+^ cDC2A (Fig. 4e), suggesting that DC-intrinsic LTβR signals function upstream of RAR signaling in the regulation of cDC2A. Similar results were observed in zDC chimeras following RA supplementation with increased frequencies of ESAM^+^ cDC2A in *Ltbr*^*+/+*^ but not *Ltbr*^*−/−*^ derived cDC2A (Fig. 4f). Moreover, administration of an anti-LTβR agonist monoclonal antibody (4H8), previously shown to augment cDC2 numbers^22^, failed to rescue cDC2 numbers in VAD or BMS-493 treated mice (Fig. 4g-h). The inability of anti-LTβR agonism to rescue cDC2 numbers in RA-deficient settings was not due to impaired LTβR expression on cDC2 since both *Ltbr* transcript and LTβR protein levels were unaffected by manipulation of RA levels (**Extended Data Fig. 6a-d**). Thus, agonism of LTβR in the absence of RA is insufficient to rescue splenic cDC2.

To determine if LTβR signaling operates to maintain cDC2A prior to or after acquisition of T-bet expression, we generated *Tbx21*^RFP-Cre^*Ltbr*^fl/fl^ mice and validated deletion of *Ltbr* in cDC2A (**Extended Data Fig. 6e**, **f**). We then generated BM chimeras using either *Tbx21*^RFP-Cre^*Ltbr*^fl/fl^ or *Tbx21*^RFP-Cre^ littermate control donor BM. Interestingly, we did not observe a difference in either cDC2A frequencies or numbers (Fig. 4i, j). To confirm these findings, we also generated zDC chimeras in which *Ltbr* was deleted in only T-bet^+^ cDC, and again observed no significant reduction in splenic cDC2A frequencies or numbers (Fig. 4k-m). In contrast, in *Zbtb4*^*Cre*^*Ltbr*^fl/fl^ mice which delete *Ltbr* in cDC2s prior to acquisition of T-bet, a significant reduction in cDC2A frequency was observed (**Extended Data Fig. 6g**, **h**), consistent with our previous competitive BM chimeras (Fig. 4a-c).

In summary, these results demonstrate a role for LTβR signaling in cDC2A development, prior to expression of T-bet.

## LTβR signaling confers preferential access to blood-proximal niches

**Fig. 5:**
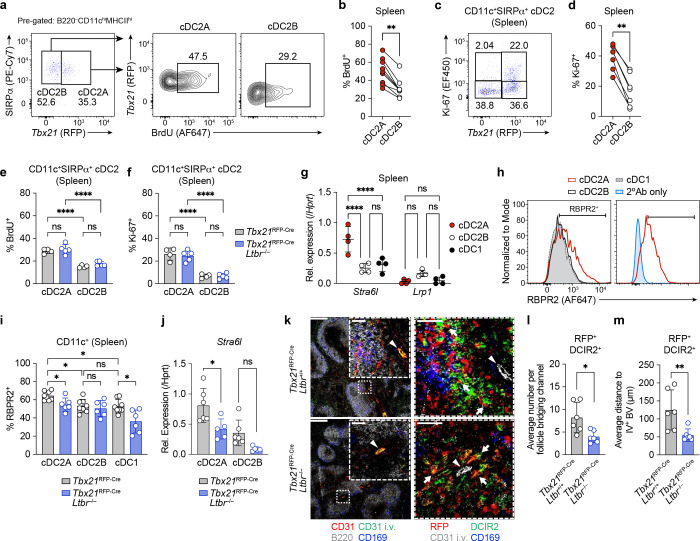
LTβR signaling confers preferential access to blood-proximal niches. **a,** Representative flow cytometry plots demonstrating incorporation of BrdU by cDC2A and cDC2B over 48h. **b,** Compiled data of **a**, lines connect data points within the same mouse (*n* = 9 per group). **c,** Representative flow cytometry plot showing Ki67 expression by cDC2A and cDC2B. **d,** Compiled data of **c**, lines connect data points within the same mouse (*n* = 7 per group). **e, f,** BrdU incorporation over 48h (**f**) and Ki67 expression (**g**) of cDC2 subsets in *Tbx21*^RFP-Cre^*Ltbr*^+/+^ (*n* = 4) or *Tbx21*^RFP-Cre^*Ltbr*^−/−^ (*n* = 5) BM chimeras. **g,** Relative expression of *Stra6l* and *Lrp1* measured by qPCR in spleen cDC subsets (n = 4 per group). **h.** Representative histograms showing RBPR2 staining intensity in splenic cDC2A (red) compared to cDC2B and cDC1. **i,** Compiled data showing RBPR2 expression in splenic cDC subsets from *Tbx21*^*RFP-Cre*^ controls (*n* = 8) and *Tbx21*^RFP-Cre^*Ltbr*^−/−^ (*n* = 6) mice. **j,** Compiled data showing *Stra6l* expression in splenic cDC subsets from *Tbx21*^*RFP-Cre*^ controls (*n* = 6) and *Tbx21*^RFP-Cre^*Ltbr*^−/−^ (*n* = 5) mice. **k,** Representative images of *Ltbr*^*+/+*^ (top) and *Ltbr*^*−/−*^ (bottom) spleen cross sections showing (left) overlap of anti-CD31 *i.v.* labeled endothelium and total CD31 staining alongside B220^+^ follicle and CD169^+^ MZ (scale bar represents 250 μm), (right) zoomed-in image of (left) showing T-bet^RFP^ DCIR2^+^ cDC2A co-localized around CD31 *i.v.* labeled endothelium. Scale bar represents 250 μm in left image and 50 μm within inset-magnified images. White triangles indicate IV^+^CD31^+^ endothelium and white arrowheads indicate identified T-bet^+^DCIR2^+^ cDC2A. **l**, **m**, Compiled data showing average number of T-bet^+^DCIR2^+^ cells per bridging channel (l) and average distance of T-bet^+^DCIR2^+^ cells from IV^+^CD31^+^ blood vessel (m), (*n* = 6 per group). Data in **b** and **d** are representative of three independent experiments; **e**, **f**, **g** are representative of one experiment; **i, j, l** and **m** are representative of two independent experiments. Statistics were measured by Wilcoxon matched-pairs signed rank tests in **b** and **d** (ns, *P*> 0.05 and ^**^P<0.01), two-way ANOVA with Sidak’s multiple-comparisons test (ns, *P*>0.05 and ^****^P<0.0001) in **e**, **f**, and **g**, and two-sided Mann-Whitney U tests in **i, j**, **l**, and **m** (ns, *P*>0.05, *P<0.05, and ^**^P<0.01). Error bars represent the mean ± s.d, each symbol represents an individual mouse.

We next explored the mechanism by which LTβR signaling promotes cDC2A differentiation within the spleen. Previous studies suggested a role for LTβR signaling in regulation of cDC2 proliferation and self-renewal ^3, 22^. In support of this, we observed elevated BrdU labelling of splenic cDC2A compared to cDC2B (~50% vs. 31% respectively) following 48hrs of BrdU administration in *Tbx21*^RFP-Cre^ mice (Fig. 5a, b). In addition, analysis of Ki67^+^ expression confirmed that cDC2A are more proliferative than cDC2B in the spleen (Fig. 5c, d), not unlike what was previously observed for unfractionated cDC2 ^3, 8, 22^. However, we did not observe differences in either BrdU incorporation or Ki67 staining in *Ltbr*^−/−^ cDC2A (Fig. 5e, f), indicating that the proliferative capacity of cDC2A is not determined by LTβR signaling.

Following dietary uptake, retinoids are packaged into chylomicrons followed by subsequent transport which is facilitated through carrier lipoproteins as well as the serum amyloid A (SAA) family of proteins and retinol-binding protein 4 (RBP4) ^24, 25^. The cell surface receptor for SAA-retinol complexes is the lipoprotein receptor-related protein 1 (LRP1) and SAA-mediated delivery of retinol to LRP1-expressing intestinal CD11c^+^ myeloid cells is required for retinaldehyde dehydrogenase expression ^25, 26^. To determine how retinol is transported to splenic cDC2s during homeostasis, we focused our attention on RBP4. Analysis of immune cells in ImmGen ^27^ as well as our scRNAseq profiling of cDC subsets across LNs revealed that *Stra6l* is most highly enriched in cDC2A (**Extended Data Fig. 7**). qPCR analysis of *Stra6l* expression in FACS-isolated splenic cDC subsets confirmed selective expression of *Stra6l* by cDC2A (Fig. 5g). We did not observe expression of *Lrp1*, an alternative retinol binding protein, in any of the cDC subsets (Fig. 5g). In addition, flow cytometry analysis demonstrated increased frequency of RBPR2^+^ cells among cDC2A relative to cDC2B and cDC1 subsets (Fig. 5h, i). Overall, these results suggest that cDC2A have enhanced capacity for retinol uptake.

Given that LTβR-deficient cDC2s fail to respond to RA, we hypothesized that LTβR expression is required for optimal access to and/or binding of Retinol/RBP4 complexes by splenic cDC2s. Notably, the frequency of RBPR2^+^ cells was reduced among *Ltbr*^−/−^ cDC2A, as well as their expression of *Stra6l*, which also trended lower in *Ltbr*^*−/−*^ cDC2B (Fig. 5i, j). To determine the location of WT vs *Ltbr*^−/−^ cDC2A within the spleen, we generated single BM chimeras using *Tbx21*^RFP-Cre^*Ltbr*^*−/−*^ or *Tbx21*^RFP-Cre^*Ltbr*^*+/+*^ donor BM and performed IMC on splenic sections. Mice were injected with anti-CD31 *i.v.* 30 minutes prior to sacrifice to identify vasculature, and analyzed as per **Extended Data Fig. 2e** with the addition of an anti-RFP antibody to identify *Tbx21*-expressing cells. We observed decreased numbers of *Ltbr*^−/−^ cDC2A in the MZBC relative to WT cDC2A (Fig. 5k, l). Moreover, *Ltbr*^−/−^ cDC2A were in closer proximity to vasculature (Fig. 5k, m). In summary, these experiments demonstrated that *Stra6l* and its corresponding RBPR2 protein are enriched in cDC2A and that LTβR signaling on cDC2s regulates positioning of cDC2A within the spleen as well as their expression of *Stra6l*/RBPR2.

## LTβR signaling regulates F-actin and splenic retention

**Fig. 6:**
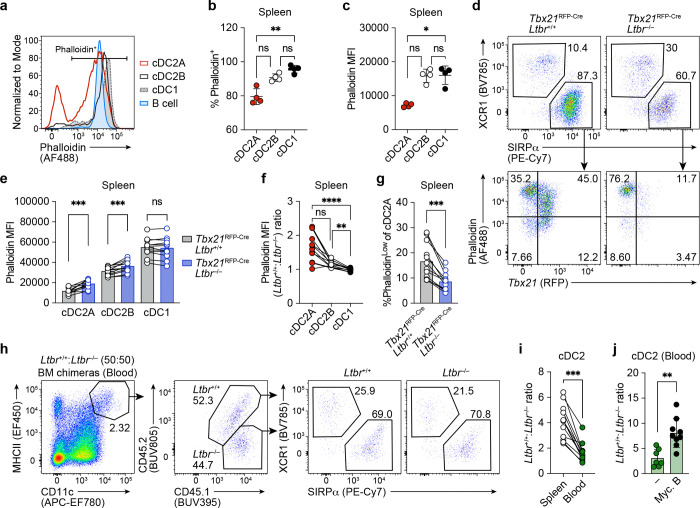
LTβR signaling regulates F-actin and splenic retention. **a,** Representative histogram depicted phalloidin staining index in cDC subsets and B cells. **b-c,** Compiled data of **a** showing (**b**) frequency of positive phalloidin-labeling in cDC subsets and (**c**) MFI of AF488-phalloidin within cDC subsets (*n* = 4 per group). **d,** Representative flow cytometry plots showing phalloidin staining of *Tbx21*^+^ and *Tbx21*^−^ cDC2 from *Ltbr*^*+/+*^(left) and *Ltbr*^−/−^ (right) DCs in a 50:50 competitive BM chimera. **e-g,** Compiled data of **d** showing (**e**) MFI of phalloidin staining in *Ltbr*^*+/+*^ and *Ltbr*^−/−^ cDC subsets in the spleens, (**f**) normalized phalloidin MFI ratios of *Ltbr*^−/−^:*Ltbr*^+/+^ in cDC subsets, and (**g**) frequency of phalloidin-low cells in the *Ltbr*^+/+^ and *Ltbr*^−/−^ splenic cDC2A compartments (*n* = 12 per group). **h**, Representative flow cytometry plots showing blood cDC from CD45.1/2^+^ *Ltbr*^*+/+*^(bottom-left) and CD45.1^+^ *Ltbr*^−/−^ (bottom-right) compartments in 50:50 competitive BM chimeras. **i**, Compiled data from **h**, comparing cDC2 chimerism (*Ltbr*^+/+^/*Ltbr*^−/−^) between the spleen and blood (n = 12 per group). **j,** DC chimerism in the blood of 50:50 (*Ltbr*^+/+^:*Ltbr*^−/−^) BM chimeras that were treated with single dose of DMSO vehicle control (*n* = 7) or Myc. B *i.v.* (1 ug) (*n* = 9) 2h post-treatment. Data in **b** and **c** are representative of one experiment; **e**-**g** and **i** are representative of two independent experiments; and **j** are representative of three independent experiments. Statistics were measured by Kruskal-Wallis test with Dunn’s multiple-comparisons test in **b**, **c,** and **f** (ns, *P*>0.05, *P<0.05, ^**^P<0.01, and ^**^P<0.01), multiple Wilcoxon paired-T tests with Holm Sidak’s adjustment (ns, *P*>0.05, and ^***^P<0.001) in **e**, Wilcoxon paired-T test in **g** and **i** (^***^, *P*<0.001), and two-sided Mann-Whitney U test in **j** (^**^, *P*<0.01). Error bars represent the mean ± s.d., each symbol represents an individual mouse.

The mis-localization of *Ltbr*^−/−^ cDC2 in the spleen prompted us to examine whether LTβR influences cDC2 motility which may affect their splenic retention and consequent access to retinol. F-actin abundance in cDC2 has previously been used as a correlative readout of DC anchoring ^28^. We therefore adapted a flow cytometry assay that uses fluorophore-conjugated phalloidin peptides to quantify intracellular F-actin content. In contrast to cDC1 and cDC2B which were uniformly F-actin high, ~20% of cDC2A expressed low levels of F-actin (Fig. 6a, b), at levels comparable to those observed in sessile cells such as pLN-derived fibroblasts (**Extended Data Fig. 8a**). Furthermore, the intensity of phalloidin staining was lower in cDC2A relative to cDC1 and cDC2B (Fig. 6c), suggesting that cDC2A are the least motile cDC subset. Examination of phalloidin staining revealed increased F-actin content in both *Ltbr*^−/−^ cDC2 subsets, most notably within cDC2As (Fig. 6d-f). Furthermore, the proportion of F-actin low cDC2As was decreased among *Ltbr*^−/−^ cDC2A relative to *Ltbr-*sufficient cDC2A from the same mouse (Fig. 6g), indicating a cDC-intrinsic role for LTβR signaling in the regulation of F-actin expression. Finally, despite the much larger reservoir of *Ltbr*^+/+^ splenic cDC2s, we observed similar proportions of *Ltbr*^−/−^ and *Ltbr*^+/+^ cDC2 in the blood, (Fig. 6h, i), suggesting escape of *Ltbr*^−/−^ cDC2 from the spleen into the blood ^28^.

To determine whether observed differences in F-actin levels resulted in egress of *Ltbr*^*−/−*^ cDC2s from the spleen, we tested whether disrupting F-actin using Mycololide B (Myc. B) toxin ^29^ would reduce the propensity of *Ltbr*^−/−^ cDC2s to accumulate in the blood. Analysis of Phalloidin expression following in vitro Myc. B treatment confirmed reduced F-actin levels in cDC2A cells, validating this approach (Extended Data Fig. **8b**, **c**). Next, we administered a single *i.v.* dose of Myc. B into *Ltbr*^+/+^:*Ltbr*^−/−^ mixed BM chimeras and observed an increase in the *Ltbr*^+/+^:*Ltbr*^−/−^ cDC2 ratio in the blood 2h post-treatment compared to vehicle-treated controls (Fig. 6j). This supports the hypothesis that the increased motility of splenic *Ltbr*^*−/−*^ cDC2s leads to their disproportionate representation in the blood.

Overall, these data show that *Ltbr*^−/−^ cDC2 cells are more motile than *Ltbr*^*+/+*^ cDC2s and are more likely to enter the circulation.

## Discussion

Although previous studies have identified heterogeneity among cDC2s, the lineage relationships between these cell types and the cues that promote cDC fates within tissues are not fully resolved. In this study, we found that nearly all cDC2s within spleen and lymphoid tissues, regardless of subset, descend from a common DC progenitor. We also showed that DC3, previously thought to be transcriptionally and ontogenically distinct from cDC2B, are transcriptionally indistinguishable from *Mgl2*^*−*^ cDC2B cells. The relative abundance of cDC2A and cDC2B subsets varied across lymphoid tissues: cDC2Bs are the dominant cDC2 subset in pLN whereas cDC2A are the dominant cDC2 subset in spleen and mLN. Interrogating the signals that determine the balance of these subsets, we found that a series of cDC-intrinsic signals in the spleen (LTβR followed by RAR signaling followed by Notch signaling) promotes the differentiation and maintenance of cDC2A identity. We did not observe cell-intrinsic RAR regulation of cDC2A in PP or pLN, suggesting that RAR signals are tissue-specific.

The existence of transcriptionally distinct cDC2 subsets raised the question of where and when these divergent fates are specified and what cues regulate their differentiation trajectories. We showed that, consistent with a recent study ^30^, cDC2A are synonymous with DC2 and that cDC2B span two subsets of cells distinguished by expression of *Mgl2*, with DC3 transcriptionally aligning with *Mgl2*^*−*^ cDC2B. Although previous studies have shown that ~40% of DC3 cells are fate mapped by *Ms4a3*-Cre ^7, 9^, indicating monocytic identity, we found that ~85% of T-bet^−^MGL2^−^ cDC2s were fate-mapped by *Clec9a*-Cre. One possibility, is that MS4A3^+^ MDPs ^31^ give rise to CLEC9A^+^ CDPs. Alternatively, monocytic DC3 may represent the fraction of non-*Clec9a* fate mapped cells among T-bet^−^MGL2^−^ cDC2s, with variability in DC3 proportions across studies reflecting differences in mice or their environments. Previous studies have attributed specific functions to MGL2/CD301b^+^ cDC2s, notably Th2-mediated immunity ^32, 33^. Thus, it is likely that MGL2^−^ and MGL2^+^ cDC2B are functionally distinct; however, elucidating the role of MGL2^−^ cDC2B will require new genetic tools.

Our finding that cDC2B subsets are derived from the classical CLEC9A^*+*^ DC progenitor agrees with a recent study of cDC2 BM progenitors ^8^. Following the initial specification of pre-cDCs into either pre-cDC2A or pre-cDC2B in the BM ^8^, our data argues that niche factors in peripheral lymphoid tissues promote the differentiation of mature cDC2A. A striking finding was the differential representation of cDC2 subsets across secondary lymphoid tissues, notably enrichment of cDC2B in pLN vs enrichment of cDC2A in mLN, PPs and spleen. These data suggest that distinct tissue cues shape cDC2 composition to ensure appropriate representation according to the likely adaptive immune responses required within these sites.

To this end, we identified two such factors, LTβR and RAR signals, and delineated the mechanisms underlying their regulation of cDC2A abundance in the spleen. Our results suggest that LTβR signaling mediates retention of cDC2A within the spleen by directly or indirectly reducing F-actin content. How splenic retention is achieved is not clear, although it may be related to the expression of integrin or adhesion molecules such as ESAM, ITGB1, ITGB2, and the cytoskeletal adaptor TALIN ^34, 35^, whose upregulation may require cDC2-intrinsic LTβR signaling ^3^. Once “settled”, CD97-CD55 mechanosensing axis and/or other mechanisms may subsequently act to retain cDC2A in the MZBC niche ^28^. Interestingly, splenic 33D1^+^ cDC2 which localize to the MZBC, are only partially labeled following *i.v.* administration of anti-CD11c antibody, suggesting a zonality of the MZBC itself ^36^. We hypothesize that this zonality may be governed by cDC2-intrinsic LTβR signaling, whereby LTβR-competent cDC2A retrieve blood-derived macromolecules such as RBP4/retinol. Indeed, we found that cDC2A uniquely express high levels of the retinol-binding protein receptor *Stra6l*/RBPR2 in a LTβR-dependent manner.

Downstream of LTβR-dependent splenic retention signals, we postulate that RAR-dependent Notch signals regulate the terminal stages of cDC2A development. This is in contrast to the role of Notch2 signaling in the differentiation of pre-DC2A progenitors in the BM^8^, suggesting temporally distinct roles for Notch2 signaling in cDC2A development. Indeed, deletion of *Notch2* using either CD11c or *Clec9a*-Cre drivers, both of which would target cDCs from the progenitor stage, result in dramatic decreases in ESAM^+^ cDC2 (cDC2A) that exceed the loss of T-bet^+^ cDC2A observed in our study following ablation of Notch2 signaling in mature T-bet^+^ cDC2A^4, 8^.

There remain questions that need answers to fill in gaps in our model. For example, we do not know the exact location of where cDC2A receive LTβR signals nor if vasculature-proximal *Ltbr*^−/−^ cDC2A are leaving or entering the spleen. Moreover, the LTβR-independent signals that support cDC2A retention in the spleen once RAR signaling is initiated in *Tbx21*-expressing cDC2A are not known. Lastly, cDC2 must presumably cooperate with an adjacent accessory cell to metabolize retinol to RA in a paracrine manner for subsequent delivery to cDC2, as splenic DCs do not possess RALDH machinery ^37, 38^. One possibility would be splenic stromal cells: indeed, WT1-expressing mesothelial stromal cells provide this function for the provision of RA to cavity macrophages, and likewise intestinal epithelial cells perform a similar function to provide RA to *Lrp1*-expressing intestinal DCs ^26, 39^. These are key questions that get to the heart of how RA rich tissue niches are generated and, warrant follow-up investigation.

Overall, our study provides new insights into cDC2 heterogeneity and tissue-specific cues that dictate the prevalence of different cDC2 subsets. Given the importance of specific cDC2 subsets for Tfh, Th2 and Th17 immunity ^1, 2, 3, 4, 11^, a better understanding of their distribution, development and maintenance will enhance our understanding of how different lymphoid tissues generate distinct types of immune responses.

## Methods

### Mice

6–8-week-old B6 and B6-Ly5.2 (CD45.1) mice were purchased from Charles River. *Zbtb46*^DTR^ (Strain:019506) and *R26*^*lsl-YFP*^ (Strain: 007903) mice were purchased from Jackson Laboratories. *Tbx21*^RFP-Cre^ mice were provided by A. Rudensky (Memorial Sloan Kettering Cancer Center). *Clec9a*^*C*re^ mice were provided by Caetano Reis e Sousa (The Francis Crick Institute). dn*Rara*^lsl/lsl^ mice express a dominant-negative form of the retinoic acid receptor RAR-α, RAR403, downstream of a *lox*P-flanked STOP cassette. These were provided by C.L. Mendelsohn (Columbia University). *Ltbr*^fl/fl^ mice were provided by J. Bromberg (University of Maryland). *Ltbr*^−/−^ CD45.1 and *Ltbr*^−/−^ CD45.2 mice were from an internal colony. *Tbx21*^RFP-CreERT2^ and *Clec9a*^Cre^ mice have been previously described ^1, 2^. *Rbpj*^fl/fl^ mice were provided by Cynthia Guidos (University of Toronto) ^3^. ^4^All mice were housed under specific-pathogen-free conditions at the Division of Comparative Medicine (DCM), University of Toronto or Sloan Kettering Institute (SKI) animal facility. Mice were analyzed between 6–12 weeks of age unless otherwise stated. Both male and female mice were included in the study, and we did not observe sex-dependent effects. All mice analyzed were age and litter matched unless otherwise specified. All animals used in this study had no previous history of experimentation and were naïve at the time of analysis. All experiments were conducted ethically under animal use protocols reviewed by the DCM and University Animal Care Committee in accordance with the Canadian Council on Animal Care, the Hospital for Sick Children’s Animal Care Committee, and the SKI Institutional Animal Care and Use Committee.

BM chimeras were generated by intravenously transferring donor cells of indicated genotypes into host animals that were lethally irradiated with two separate doses of 550 rad of γ-irradiation given 4h apart. Recipient mice were given neomycin sulfate (2 g/L, Bioshop Canada) in their drinking water for the first two weeks post-irradiation and left for an additional 6–8 weeks before experimentation to ensure full reconstitution of the immune system. For ablation of *Zbtb46*-expressing cells in mixed BM chimeras, a first dose of diphtheria toxin (DT) (Sigma-Aldrich, D0564) was administered intraperitoneally at 20 ng/g body weight followed by subsequent doses of 4 ng/g DT every three days to maintain DC ablation. DT treatment was maintained for 10 days with the mice being euthanized 12–24h after the last DT treatment as described previously by Meredith et al ^5^.

*In vivo* labeling of blood-associated endothelium in the spleen was performed by intravenously administering 10 μg of APC-conjugated anti-CD31 (390, Biolegend) for 30 min prior to animal euthanasia, as previously described by Wu et al ^6^. *In vivo* inhibition of F-actin was performed by intravenously administering 1 μg of Mycalolide B^7^ (provided by John Allingham, Queen’s University at Kingston, Canada).

### DC isolation from lymphoid tissues and blood

Spleens were mashed into single cell suspensions prior to digestion. Cell suspensions were then digested in Hank’s Buffered Saline Solution (with 10 mM HEPES (Gibco), 150 mM NaCl, 5 mM KCl, 1mM MgCl_2_, 1.8 mM CaCl_2_ added, final pH 7.2–4) using 1 mg/ml Collagenase D (Roche) and 0.2 mg/ml DNase I (Thermofisher) for 45 min at 37°C with continuous agitation. 1 mM EDTA was added to solution to stop digestion for 10 min at 20°C. PP and mesenteric lymph node (mLN) tissues were minced with a surgical blade and then digested using 1 mg/ml Collagenase P (Sigma-Aldrich) (2 mg/ml was used for PP tissues) and 0.1 mg/ml DNase I (Sigma-Aldrich) in complete media (10% FBS, 10 mM HEPES, 1% penicillin/streptomycin (Sigma-Aldrich), 1 mM L-glutamine (Sigma-Aldrich) for 40 min at 37°C with continuous agitation. After digestion, LN and PPs were gently pipetted up and down until tissues dissolved into a homogenous solution. Blood was collected from mice terminally using a cardiac puncture with a 25G needle into Microvette^®^ Lithium heparin capillary tubes. RBCs were lysed using eBioscience 1X RBC Lysis Buffer for 4 min. Finally, all cell suspensions were washed using PBS and filtered through a 70 μm strainer.

### Flow Cytometry

Cell suspensions were isolated from tissues, as described above, and blocked with 2.4G2 antibody (generated in-house from B cell hybridomas and purified using protein G affinity chromatography beads, used at 1 μg/10^6^ cells). For antibody staining, the following reagents were used in PBS supplemented with 2% fetal bovine serum: B220 (RA3–6B2), BrdU (MoBU-1), CD8a (53–6.7), CD11b (M1/70), CD31 (390), CD45.1 (A20), CD45.2 (104), CD117 (2B8), CD135 (A2F10), Epcam (OX-7), Ki67 (SolA15), LTβR (3C8), Rabbit IgG (Polyclonal), Ly6C (HK1.4), MHC II (I-A/I-E), and TER-119 (TER-119) from Thermofisher; CD5 (53–7.3), CD11c (N418), CD43 (S11), CD170 (S17007L), Clec10A (LOM-14), ESAM (1G8), Ly6D (49-H4), Podoplanin (8.1.1), SIRPa/CD172 (P84), CD301b/Mgl2 (URA-1) and XCR1 (ZET) from Biolegend; RBPR2 (Polyclonal) from St. John’s Laboratory; and SiglecH (551) from BD Biosciences. Dead cells were excluded using LIVE/DEAD^™^ Fixable Aqua Viability Dye (Thermofisher) and non-singlet events were removed using FSC-H/W characteristics. Intracellular staining was enabled by using the Cytofix/Cytoperm Kit (BD Biosciences). For detection of intracellular F-actin content, cells were stained with 37.5 mM AF488-conjugated Phalloidin (Thermofisher) for 1h at 4°C. All data was acquired on a X20 Fortessa^™^ (BD Biosciences) or BD FACSymphony^™^ A3 using BD FACSDiva^™^ (BD Biosciences) and a combination of the following lasers and filters: R639 (670/30, 780/60), YG561 (586/15, 780/60), B488 (525/50, 710/50), V405 (450/50, 525/50, 610/20, 710/50, 780/60), and UV349 (379/28, 515/30, 610/25, 670/25, 810/40). Subsequent data analysis was conducted using FlowJo V.10 software (Treestar).

### In-vivo modulation of RAR signaling

Chronic inhibition of RAR signaling was achieved by feeding pregnant dams (pups at E14) with a Vitamin A Deficient diet (TD.10991, Envigo) or Vitamin A Control diet (TD.10992, Envigo). Diets were continued post-birth and post-weaning until mice reached 8–10 weeks of age for analysis. Acute inhibition of RAR signaling was achieved by intraperitoneal administration of 220 μg BMS493 (Tocris) daily or DMSO vehicle control over a 9-day regimen. Exogenous RA supplementation to enhance or rescue RAR signaling was performed by intraperitoneal administration of 250 μg all-trans RA (Tocris) over a 10-day regimen or as otherwise indicated in the experiment. Pharmacological reagents were stored in DMSO for long-term storage and subsequently diluted in PBS for *in vivo* administration.

### Cell sorting of DC subsets

For sorting of purified cDC subsets, tissues were isolated as described above. CD11c^+^ cells were enriched from cell suspensions using the EasySep^™^ CD11c Positive Selection Kit II (Stem Cell) as per manufacturer’s instructions. An APC-conjugated CD11c antibody (N418, 1 ug/ml) was added alongside CD11c selection cocktail to fluorescently label surface CD11c. Staining for the additional surface markers and for viability was subsequently performed on CD11c-enriched cells and cDC subsets were subsequently sorted on a FACSAria^™^ IIIu (BD Biosciences). Sorted cells were collected into 50/50% PBS/FBS solution or directly into RLT lysis buffer provided by Qiagen^™^.

### BrdU labeling

To track proliferation of immune cells over a 48h duration, 5 bromo-2’-deoxyuridine (BrdU) was injected into mice at a dose of 10 mg/kg intraperitoneally 48h, 24h, and 1h before euthanization. In between doses, mice were also continuously fed drinking water containing 0.75 mg/ml BrdU. Cells were stained intracellularly for incorporated BrdU using the eBioscience^™^ BrdU Staining Kit (Thermofisher) as per the manufacturer’s instructions, using Alexa Fluor 647 anti-BrdU antibody (Thermofisher).

### LTβR Agonism

αLTβR agonist antibodies (rat IgG anti-mouse LTβR clone 4H8) and anti-HEL control antibodies were received as a gift from C. Ware (Sanford Burnham Prebys Institute). αLTβR antibodies were administered intraperitoneally into animals at a daily dose of 100 μg thrice at days 0, 3, and 6 before euthanizing mice on day 9 for analysis. Control animals were given equal dose of α-Hel isotype control antibodies.

### Mouse scRNA sequencing

Mouse DC subsets were enriched from a pool of mesenteric lymph nodes or spleen, obtained from four *Clec9a*^*Cre/Cre*^*Tbx21*^*RFP-CreERT2*^*R26*^*lsl-YFP*^ mice. Lymphoid organs were digested in RPMI-1640 supplemented with 5% fetal calf serum, 1% L-glutamine, 1% penicillin–streptomycin, 10 mM HEPES, 1 mg ml−1 collagenase A (Sigma, 11088793001) and 1 U ml−1 DNase I (Sigma, 10104159001)(final pH = 7.2–4) for 45 min at 37 °C, 250 rpm. Digested samples were filtered through 100-μm strainers and centrifuged to remove collagenase solution. Cells were depleted of Lin+ (TCRb, TCRγδ, CD19, Ter-119, NK1.1)^+^ cells by staining with biotinylated antibodies followed by magnetic bead negative selection (M-280 dynabeads; ThermoFisher). Cells were incubated with anti-CD16/32 in sorting buffer (2% FBS in PBS) for 10 min at 4°C to block binding to Fc receptors. Extracellular antigens were stained for 30 min at in sorting buffer. mLN and spleen samples were separately “stained” with different TotalSeq^™^ oligo-conjugated antibodies consisting of a pool of antibodies directed against CD45 (30-F11) and H2 MHC Class I alloantigens (clone M1/42; Biolegend). Cells were washed and resuspended in sorting buffer with SYTOX blue (Invitrogen) for exclusion of dead cells. Live, Lin (CD19, NK1.1, TCRb, SiglecF)^−^MHCII^+^YFP^+^ or YFP^−^ cells were sorted into complete RPMI-20% FBS, before being pelleted and resuspended in complete RPMI-2% FBS. YFP^+^ and YFP^−^ cells were combined as follows: 1,600 mLN and 13,400 splenic YFP^+^ cells, 9,700 mLN and 5,300 splenic YFP^−^ cells. Cells were stained with Trypan blue and Countess II Automated Cell Counter (ThermoFisher) was used to assess both cell number and viability. Following QC, the single cell suspensions were loaded onto Chromium GEM-X 3’ Chip (10x Genomics PN 1000690) and GEM generation, cDNA synthesis, cDNA amplification, and library preparation of 2,300–3,800 cells proceeded using the Chromium GEM-X Single Cell 3’ Kit v4 (10X Genomics PN 1000691) according to the manufacturer’s protocol. cDNA amplification included 11 cycles and 13–35 ng of the material was used to prepare sequencing libraries with 10 cycles of PCR. Indexed libraries were pooled equimolar and sequenced on a NovaSeq X in a PE28/88 paired end run using the NovaSeq X 10B Reagent Kit (100 cycles) (Illumina). An average of 31 thousand paired reads was generated per cell. Amplification products generated using the methods described above included both cDNA and feature barcodes tagged with cell barcodes and unique molecular identifiers. Smaller feature barcode fragments were separated from longer amplified cDNA using a 0.6X cleanup using aMPure XP beads (Beckman Coulter catalog # A63882). Libraries were constructed using the 3’ Feature Barcode Kit (10X Genomics PN 1000276) according to the manufacturer’s protocol with 10 cycles of PCR. Indexed libraries were pooled equimolar and sequenced on a NovaSeq X in a PE28/88 run using the NovaSeq X 10B Reagent Kit (100 cycles) (Illumina). An average of 421 million paired reads was generated per sample. FASTQ files were aligned to mm10 (Cell Ranger mouse reference genome mm10–2020-A) and counted by Cell Ranger v9.0.1 with default parameters. HTO sequencing data were aligned to the HTO barcodes, and UMIs were counted for each cell using CITE-seq-Count. Samples were then demultiplexed using the Cell Ranger Multi pipeline ^8^.

### Mouse single-cell RNA-seq computational analysis

#### Pre-processing of the 10X scRNA-seq dataset for dendritic cells:

The singlet barcodes were further filtered based on the number of RNA-seq transcripts (>400), the number of detected genes (>250), and the fraction of mitochondrial transcripts (<8%). Finally, any genes detected in <2 cells in the scRNA-seq data were discarded, leaving 19,358 genes. After clustering the scRNA-seq data (described in ‘Dimensionality reduction, cell clustering, and visualization’), we identified 13 contaminant clusters spanning B cells, plasma cells, neutrophils and ILC3 and three low quality clusters which were excluded from downstream analyses. In total, 2,198 cells remained, with a median scRNA-seq library-size of 12,382. Cluster labels were manually assigned and curated based on expression of canonical genes, differentially expressed genes identified by MAST analysis ^9^ (described in ‘differential gene expression tests’) as well as cell type annotations assigned by SCimilarity ^10^.

#### Dimensionality reduction, cell clustering, and visualization:

Following quality control filtering, raw counts were library-size normalized, log transformed (‘log-normalized’ expression values) and scaled using Scanpy v1.10.3. A nearest-neighbour graph was constructed using the top 40 principal components (PCs) with 25 nearest neighbours. Clustering was performed using PhenoGraph^11^ (k=25, clustering_algo = ‘louvain’) generating 30 clusters. Cells annotated as pDC, cDC1, cDC2A, cDC2B, CCR7^+^ DC, monocytes/macrophages, were re-clustered with PhenoGraph (k=25, clustering_algo = ‘louvain’) generating 16 clusters. We identified one minor contaminant cluster (TC I) and five low quality clusters which were excluded from downstream analyses. Cell clustering was visualized using UMAP ^12^, computed from the nearest neighbour graph built by PhenoGraph.

#### Differential gene expression tests:

Differentially expressed genes (DEGs) between groups of cells were identified with MAST ^9^, performed using Seurat functions. MAST was run on the log-normalized expression values. In all tests, genes were only considered if they were detected in at least 1% of the cells in at least one of the two groups compared (min.pct=0.01, logfc.threshold=0). In one-vs-rest DE tests comparing multiple groups, each group was compared to all the cells from other groups. Specific DE comparisons are described in the results. DEGs were reported according to their log-fold change (>1.5) and adjusted p-value (<0.01). The top DEG markers were subsequently selected for each group, based on fold change and p-value.

#### Data imputation:

MAGIC imputation^13^ was applied to the log-normalized expression values to de-noise and recover missing values. Imputed data were used only for visualization of gene expression on heatmaps where specified.

#### Analysis of publicly available dendritic cell scRNA-seq datasets:

scRNA-seq profiles of splenic dendritic cells were downloaded from publicly available datasets (GSE262474, GSE137710, and figshare https://doi.org/10.6084/m9.figshare.22232056.v1).^14, 15,16^. For Brown et al. (GSE262474) and Liu et al. (figshare), cluster cell type annotations were available, and no reanalysis of the data was performed. For the scRNA-seq datasets from Rodrigues (GSE262474), each sample was first demultiplexed based on HTO counts using HTODemux function in Seurat v4.4.0. Next, RNA count matrices for the cell barcodes classified as singlets from demultiplexing were merged into a single count matrix.The RNA count matrix was further filtered based on the number of transcripts (>2,000 and <80,000), the number of detected genes (>1000 and <7,000), and the fraction of mitochondrial transcripts (<5%). The filtered count matrix was library-size normalized, log-transformed (‘log-normalized’ expression values) and then centered and scaled (‘scaled’ expression values) using Seurat v4.4.0. Any genes detected in <2 cells were discarded. Principal component analysis (PCA) was performed on the scaled data (npcs=50). A nearest-neighbor graph was constructed using the first 30 principal components (PCs) with 30 nearest neighbors. Clustering was performed using Louvain algorithm (resolution=0.8) on the shared nearest-neighbor graph. Cell clustering was visualized using UMAP computed from the same nearest neighbor graph used for clustering. Cluster labels were manually assigned and curated based on expression of canonical genes, differentially expressed genes identified by MAST analysis. Clusters annotated as dendritic cells or monocyte/macrophages were selected for downstream integration.

#### Integration of dendritic cell scRNA-seq datasets:

The four scRNA-seq datasets were integrated using Seurat’s anchor-based integration method. First, 5,000 genes were first selected based on their repeated variability across datasets to identify anchors using canonical correlation analysis. The integration of the datasets was then performed using ‘IntegrateData’ function. Next, the expression values of genes in the integrated dataset were scaled and used for PCA. A UMAP embedding of the integrated dataset was computed based on the top 30 principal components and used for visualization of the cells.

### RT-PCR

Total RNA was isolated using a Qiagen^™^ RNeasy Micro Kit as per manufacturer’s instructions from flow-sorted cDC subsets as described above. RNA was converted into cDNA using Superscript IV Reverse Transcriptase kit (Thermofisher). RT-PCR was performed with SYBR Green Master Mix (Thermofisher) on a CFX384 Touch^™^ Real-Time PCR Detection System (BioRad). Primer sets are listed below and were generated by IDT DNA Technologies:

*Hprt* forward: CCC CAA AAT GGT TAA GGT TGC

reverse: AAC AAA GTC TGG CCT GTA TCC

*Lrp1* forward: GGA CCA CCA TCG TGG AAA

reverse: TCC CAG CCA CGG TGA TAG

*Rbpr2* forward: TCC TGG GGA ACC ACT TTG GA

reverse: CCA TGC CTC CGA TGA AAA GC

*Ltbr* forward: CCA GAT GTG AGA TCC AGG GC

reverse: GAC CAG CGA CAG CAG GAT G

*Dtx1* forward: TGG AAC GAG ATT CAC CAC AA

reverse: CTC AGC CAG CAC GTT GTC TA

*Hes1* forward: TCC TGA CGG CCA ATT TGC

reverse: GGA AGG TGA CAC TGC GTT AGG

### Imaging Mass Cytometry (IMC) Acquisition and Analysis

Spleen tissues were fixed in 4% PFA for 1h and embedded in 30% sucrose overnight. Histological analysis was performed on 7 μm frozen spleen sections that were fixed in acetone for 7 min at −20°C. Sections were rehydrated in PBS and non-specific protein interactions were blocked using Super Block^™^ solution (Sigma). For imaging mass cytometry, slides were stained for 1h at 20°C or at 4°C overnight with assay-dependent concentrations of metal-conjugated antibodies (**Supplementary Table 2**). For the *Ltbr*^+/+^ and *Ltbr*^*−/−*^ cohorts, image acquisition was carried out on the Standard BioTools (formerly Fluidigm) Hyperion XTi Imaging System following manufacturer’s instruction at a laser frequency of 800 Hz using CYTOF^™^ software version v9.2.1 and step size of 1 μm. Images were taken at a resolution of 1 μm/pixel using cell mode. Regions of interest ranging from 1000 μm × 1000 μm to 1,500 μm × 1,500 μm were selected based on bright-field images. 2–3 regions of interest were collected per speen section to ensure accurate representation. For the VAD cohort, image acquisition was carried out on the Standard BioTools (then Fluidigm) Hyperion Imaging System following manufacturer’s instruction at a laser frequency of 200–400 Hz using CYTOF^™^ software version v5.1.602 software. Images were acquired at a resolution of 1 μm/pixel, step size of 1 μm and ablation energy of 5–6Db. Regions of interest ranging from 1500 μm × 1500 μm to 2000 μm × 2000 μm were selected based on bright-field images

For IMC analysis, merged images of appropriate channels to identify follicles and bridging channels (CD3, B220, CD169), cDC2A populations (identified as CD3^−^B220^−^Ly6G^−^CD11c^+^DCIR2^+^T-bet-RFP^+^), and blood-adjacent endothelium (CD31^+^CD31-IV^+^) were Created in MCD Viewer. Average DCIR2 intensity in the bridging channel, number of cDC2A in the bridging channel, and distance to closest IV^+^ blood vessel, and follicle and PALS sizes were measured in ImageJ v1.54 using the contour selection tool or line selection tool at a global scale of 1 pixel = 1 μm.

Raw 16-bit ome.tiff files were generated from MCD files or corresponding .txt files. Data were passed through the Percentile Normalization GUI Image deNoising (PENGUIN v0.1.1) pipeline ^17^. Cell masks were constructed for each ROI using a combination of CD45, CD11c, CD3, B220, and CD11b surface staining using the Mesmer algorithm ^18^, available in the steinbock IMC analysis toolkit (v.016.3) ^19^. Per cell intensities were measured for each channel using the steinbock toolkit and loaded into Rstudio (v2023.03.0) for single-cell analysis using SingleCellExperiment (v1.20.0), Seurat (v5.3.0), and SpatialExperiment (v.1.10.1) packages.

### Statistics

Statistical analysis and graphing were performed using Prism (GraphPad) software. Analysis of all data was done with unpaired two-tailed t test, one or two-way ANOVA with a 95% confidence interval, two-sided Mann-Whitney U non-parametric test (unpaired) or a Kruskal-Wallis test (paired), as specified in the text or legends. *P* < 0.05 was considered significant, after correcting for multiple comparisons: * *p* < 0.05; ** *p* < 0.01; ***p < 0.001; ****p < 0.0001. Details as to number of replicates, sample size, significance tests, and value and meaning of *n* for each experiment are included in the Methods or Figure legends. Statistical tests were performed with Prism (GraphPad Software). scRNA-seq experiments were carried out once. Mice were non-randomly allocated to experimental groups to ensure equal distribution of genotypes between treatments. Researchers were not blinded as to genotype or treatment during the experiments. No measures were taken to estimate sample size of to determine whether the data met the assumptions of the statistical approaches used.

### Reporting summary

Further information on research design is available in the Nature Portfolio Reporting Summary linked to this article.

## Supplementary Material

Supplementary Files

This is a list of supplementary _les associated with this preprint. Click to download.

• SupplementaryTable2.csv

• SupplementaryTable1.csv

## Data Availability

The mouse sequencing data are available through the Gene Expression Omnibus under accession number GSE302089. This manuscript makes use of previously published scRNA-seq data from GSE262474, GSE137710 and figshare https://doi.org/10.6084/m9.figshare.22232056.v1.
